# Macrophage Immunometabolism in Pulmonary Homeostasis and Chronic Lung Diseases

**DOI:** 10.7150/ijbs.123492

**Published:** 2025-10-10

**Authors:** Cong Xie, Maimaititusun Yalikun, Zhenhui Ruan, Hang Yu, Xi Huang, Huahe Zhu, Wenglam Choi, Qingli Luo, Zhen Gao, Jingcheng Dong

**Affiliations:** 1 Institutes of Integrative Medicine, Fudan University, Shanghai 200032, China.; 2 Yuquan Hospital, School of Clinical Medicine, Tsinghua University, Beijing 100040, China.; 3 College of Traditional Chinese Medicine, Xinjiang Medical University, Urumqi 830017, China.; 4 Shanghai Institute of Infectious Disease and Biosecurity, Fudan University Shanghai Medical College, Shanghai 200032, China.; 5 Charité - Universitätsmedizin Berlin, corporate member of Freie Universität Berlin and Humboldt-Universität zu Berlin, Berlin 10117, Germany.; 6 Department of Biochemistry, Faculty of Medicine and Dentistry, University of Alberta, Edmonton T6G 2H7, Canada.; 7 Shanghai Pulmonary Hospital, Tongji University School of Medicine, Shanghai 200433, China.; 8 Department of Integrative Medicine, Huashan Hospital Affiliated to Fudan University, Shanghai 200040, China.

**Keywords:** macrophage immunometabolism, pulmonary homeostasis, chronic lung diseases, alveolar macrophages, metabolic reprogramming, therapeutic targets

## Abstract

Macrophages play a central role in maintaining pulmonary immune homeostasis and responding to injury. In the lung, alveolar macrophages modulate their metabolic profiles to support essential functions such as microbial clearance, inflammation resolution, and tissue repair. Recent studies have shown that these metabolic adaptations are not merely byproducts of activation but represent key regulators of macrophage behavior. In chronic lung diseases including asthma, chronic obstructive pulmonary disease (COPD), and idiopathic pulmonary fibrosis (IPF), macrophage metabolism is pathologically reprogrammed, contributing to persistent inflammation in asthma and COPD, or to unrestrained fibrotic remodeling in IPF, and ultimately leading to ongoing tissue damage. Specifically, in asthma, type 2 cytokine signaling promotes alternative macrophage activation, accompanied by increased fatty acid oxidation and disrupted lipid mediator profiles. COPD-associated macrophages exhibit mitochondrial dysfunction, enhanced glycolysis, and iron overload, impairing bacterial phagocytosis and amplifying oxidative stress. In IPF, macrophages simultaneously engage glycolytic and oxidative pathways while losing regulatory metabolites such as itaconate, supporting persistent fibrogenic signaling. These disease-specific metabolic features sustain maladaptive macrophage phenotypes and constitute promising targets for therapeutic intervention. This review outlines current knowledge of macrophage immunometabolism in the lung and its contribution to chronic respiratory diseases. It also discusses strategies to restore metabolic balance, including the use of antioxidants, metabolic modulators, and targeted drug delivery. Understanding macrophage metabolism may open new avenues for treating chronic lung diseases at the level of cellular function.

## 1. Introduction

**Figure 1:**
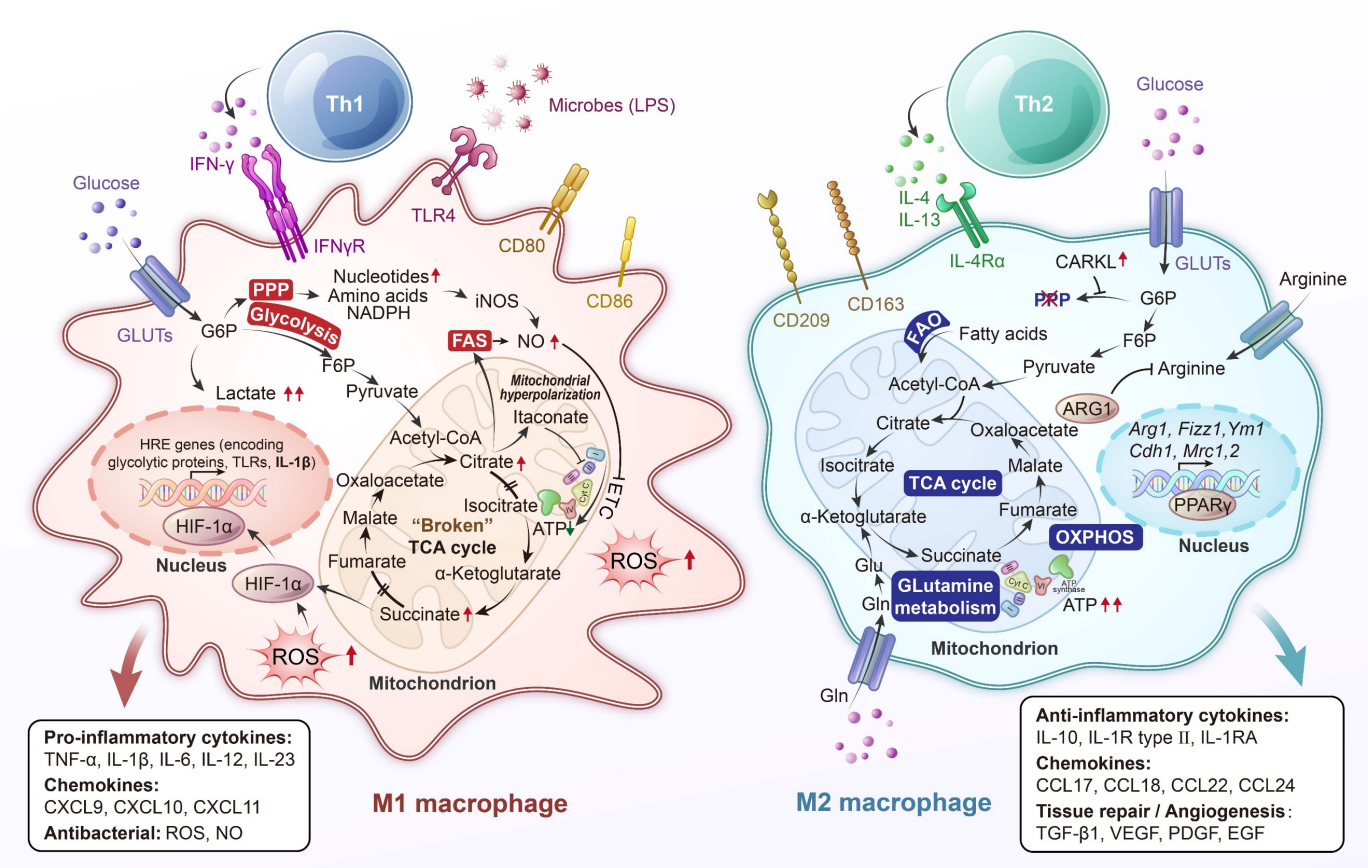
** Metabolic rearrangement in macrophage polarization to classically activated proinflammatory “M1” macrophages or alternatively activated anti-inflammatory “M2” macrophages.** Pro-inflammatory stimuli induce the activation of specific pathways through the stabilization of transcription factors such as nuclear factor (NF)-κB and hypoxia-inducible factor (HIF)-1α, which trigger the expression of markers like inducible nitric oxide synthase (iNOS), CD80, and CD86 and the release of tumor necrosis factor (TNF)-α, IL-1β, IL-6, IL-12, and IL-23. Cells undergo a metabolic reprogramming toward glycolysis, the pentose-phosphate pathway, and fatty acid synthesis. This associates to interruption of tricarboxylic acid (TCA) cycle, ROS formation and efflux of citrate, which supports nicotinamide adenine dinucleotide phosphate (NADPH) and prostaglandin (PG) E_2_ synthesis, and succinate, which stabilizes HIF-1α, thereby promoting lipopolysaccharide (LPS)-induced expression of IL-1β. Alternatively, anti-inflammatory “M2” macrophages are induced by cytokines such as IL-4 and IL-13, leading to activation of transcription factors like STAT6 and peroxisome proliferator-activated receptor-γ (PPAR-γ). These cells are characterized by the expression of markers such as arginase-1 (Arg1), CD206 (mannose receptor), and Ym1, and the secretion of anti-inflammatory mediators like IL-10 and TGF-β. M2 polarization is associated with enhanced oxidative metabolism, including increased fatty acid oxidation (FAO) and mitochondrial oxidative phosphorylation (OXPHOS), along with an intact TCA cycle. This metabolic profile supports cellular functions such as tissue repair, matrix remodeling, efferocytosis (clearance of dead cells), and resolution of inflammation. Additionally, metabolic regulators like carbohydrate kinase-like protein (CARKL) and PPAR-γ coactivator 1 β (PGC-1β) contribute to maintaining redox balance and limiting pro-inflammatory responses. ATP, adenosine triphosphate; ETC, electron transport chain; FAS, fatty acid synthesis; F6P, fructose-6-phosphate; Gln, glutamine; Glu, glutamic acid; GLUT, glucose transporter; G6P, glucose-6-phosphate; HRE, hypoxia response element; IFN-γ, interferon-γ; PPP, pentose phosphate pathway; TLR4, toll-like receptor 4.

Lung-resident macrophages play a central role in pulmonary immune defense and the maintenance of tissue homeostasis 1. Alveolar macrophages, which are the predominant immune cells in the healthy lung, act as frontline sentinels that continuously clear inhaled particles and microbes while regulating inflammation to preserve the integrity of the gas-exchange surface. A defining characteristic of macrophages is their immunometabolism, the close integration between cellular metabolic pathways and immune functions. Pro-inflammatory (“M1”) activation, such as stimulation by lipopolysaccharide (LPS), typically induces a shift from oxidative phosphorylation (OXPHOS) to aerobic glycolysis, even in the presence of sufficient oxygen, a phenomenon reminiscent of the Warburg effect observed in cancer cells 2. In contrast, exposure to anti-inflammatory (“M2”) signals like interleukin (IL)-4 promotes mitochondrial respiration and fatty acid oxidation (FAO) 3. These metabolic programs are not just consequences of activation states but actively shape macrophage behavior, including cytokine production, antimicrobial capacity, and tissue-repair functions (**Figure 1**) 4.

It is widely accepted that macrophage activation is driven by cytokines present in infected or damaged tissues 5. However, recent findings suggest that metabolic adaptation itself also plays a critical and instructive role in determining macrophage functional identity 6. Rather than being a passive byproduct, metabolic state can influence intracellular signaling, epigenetic remodeling, and effector differentiation. Within the lung, local environmental conditions such as high oxygen tension, limited glucose availability, and the presence of surfactant lipids impose unique metabolic constraints on resident macrophages 7, 8. Under chronic disease conditions such as asthma, chronic obstructive pulmonary disease (COPD), and idiopathic pulmonary fibrosis (IPF), these macrophages undergo profound metabolic reprogramming as they adapt to inflammatory and remodeling signals in their microenvironment 9. These metabolic changes can support protective functions such as microbial clearance and debris removal, but they may also contribute to pathological inflammation, tissue injury, and fibrosis 10.

Specific metabolic signaling pathways act as crucial bridges between cellular metabolic state and immune function. In M1 macrophages, for example, accumulation of the tricarboxylic acid (TCA) intermediate succinate stabilizes hypoxia-inducible factor (HIF)-1α, which shifts metabolism toward glycolysis and drives the production of IL-1β 11. In parallel, the energy-sensing kinase AMP-activated protein kinase (AMPK) is activated by cellular stress or anti-inflammatory signals such as IL-10, promoting oxidative metabolism while inhibiting the mechanistic target of rapamycin (mTOR) pathway and thereby enhancing an anti-inflammatory macrophage phenotype 12. By contrast, mTOR complex 1 (mTORC1) supports anabolic metabolism and facilitates translation of pro-inflammatory cytokines, including the optimal synthesis of IL-1β in classically activated (“M1”) macrophages 13. Nutrient and oxygen sensors therefore tightly regulate macrophage polarization: HIF-1α favors a glycolytic, pro-inflammatory program 14, whereas peroxisome proliferator-activated receptor-γ (PPAR-γ) and AMPK tilt metabolism toward FAO and encourage anti-inflammatory, tissue-reparative functions 12. Through these pathways, metabolic adaptation not only results from external activation signals but also feeds back to shape them, creating a bidirectional link between a macrophage's metabolic state and its immune effector profile.

In this review, we examine how macrophage immunometabolism is altered in chronic lung diseases and the implications of these changes for disease progression. We begin by describing the metabolic features of lung macrophages under homeostatic conditions, followed by a focused analysis of disease-specific metabolic reprogramming in asthma, COPD, and IPF. We also highlight emerging therapeutic strategies aimed at restoring healthy macrophage metabolism. A deeper understanding of these mechanisms may provide novel opportunities for targeted treatment of chronic respiratory diseases.

## 2. Lung-resident macrophages and their heterogeneity, functional plasticity, and metabolic phenotype at homeostasis

**Figure 2:**
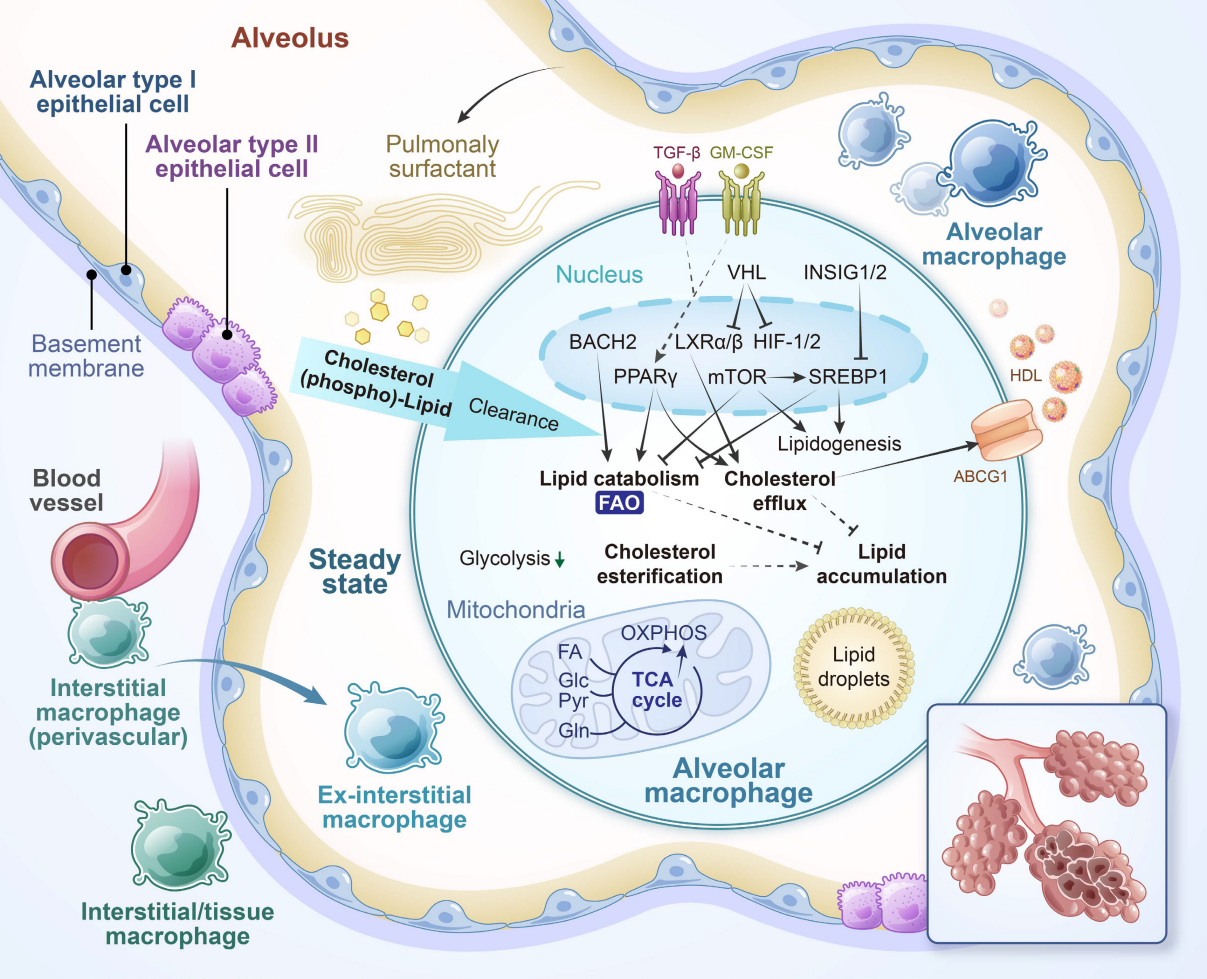
** Functional and metabolic characteristics of alveolar macrophages (AMs) under homeostatic conditions.** Under steady-state conditions, AMs maintain immune tolerance and regulate pulmonary surfactant—a lipoprotein complex produced by respiratory epithelial cells to lubricate the lungs and facilitate frictionless expansion/contraction—through anti-inflammatory signaling and lipolytic metabolism. Equipped with robust phagocytic capacity, AMs efficiently clear apoptotic/senescent cells and inhaled particles, serving as the first line of defense against airborne pathogens. Their energy metabolism primarily relies on oxidative phosphorylation (OXPHOS) and fatty acid oxidation (FAO), a process governed by peroxisome proliferator-activated receptor-γ (PPARγ)-driven transcriptional programs and modulated by epithelial-derived signals such as granulocyte-macrophage colony-stimulating factor (GM-CSF). Impaired cholesterol handling, including deficiencies in liver X receptor (LXR) and its downstream transporter ABCG1 (ATP-binding cassette subfamily G member 1), can lead to pathological surfactant accumulation, as observed in pulmonary alveolar proteinosis. Furthermore, oxygen-sensing pathways such as the von Hippel-Lindau (VHL)-HIF axis shape AM identity and metabolic states, highlighting the critical role of tightly regulated lipid metabolism in maintaining pulmonary homeostasis. BACH2, BTB and CNC homology 2; HDL, high-density lipoprotein; INSIG, insulin-induced gene; SREBP1, sterol regulatory element-binding protein 1. This figure was substantially revised and repurposed based on Figure 2A from Wculek SK et al., Cell Mol Immunol. 2022;19:384-408.

The lung harbors multiple populations of macrophages that differ in developmental origin, anatomical localization, and function 15. Among them, alveolar macrophages (AMs) are located in the alveolar space, where they continuously clear inhaled particles, surfactant components, and apoptotic cells. Interstitial macrophages (IMs) are situated in the lung parenchyma, often adjacent to blood vessels and airways, and are more involved in modulating immune responses and maintaining tissue homeostasis (**Figure 2**) 16.

Under physiological conditions, most AMs originate from embryonic precursors and maintain themselves through local proliferation 17, 18. In fact, the majority of tissue-resident macrophages in adults are seeded before birth, arising from yolk sac- and fetal liver- derived progenitors during embryogenesis. Fate-mapping/lineage-tracing approaches, parabiosis experiments, and adoptive transfer studies have established that AMs sustain their population throughout life primarily via self-renewal, with minimal contribution from adult bone marrow-derived monocytes 19. This long-lived, self-maintaining population is supported by factors such as granulocyte-macrophage colony-stimulating factor (GM-CSF) and transforming growth factor (TGF)-β 20. When the lung is injured or inflamed, circulating monocytes can be recruited to the alveoli and differentiate into monocyte-derived AMs (Mo-AMs) 21. These newly differentiated cells initially exhibit transcriptional and metabolic profiles that differ from those of long-lived resident AMs 22-24. For example, they tend to be more pro-inflammatory and rely more on glycolysis during early stages of adaptation 24, 25. Over time, monocyte-derived macrophages may gradually adopt phenotypic features of resident AMs 26, although some differences can persist, particularly under chronic disease conditions 27.

The pulmonary environment imposes distinct metabolic demands on resident macrophages. The alveolar space is characterized by high oxygen levels, relatively low glucose availability, and abundant lipids derived from pulmonary surfactant 28. In response to these conditions, AMs predominantly utilize FAO and OXPHOS to meet their energy requirements. At baseline, they exhibit low glycolytic activity and depend on mitochondrial respiration fueled by lipid catabolism. This metabolic profile is regulated by transcription factors such as PPAR-γ, which promotes lipid uptake and mitochondrial activity, and is induced by GM-CSF signaling 29. These pathways support both surfactant clearance and the immunoregulatory phenotype of AMs. IMs, on the other hand, display more heterogeneity in both function and metabolism, reflecting their distribution across different interstitial niches 30. Although less well-characterized, IMs are thought to engage more variable metabolic programs, including glycolysis and glutaminolysis, depending on local environmental cues.

Metabolism is not simply a reflection of macrophage activation; rather, it plays a directive role in shaping immune function (**Table 1**) 6. Inflammatory stimuli such as LPS trigger a shift toward glycolysis and a disrupted TCA cycle to support cytokine production and antimicrobial activity. In contrast, anti-inflammatory signals like IL-4 promote a metabolic state characterized by intact TCA activity and enhanced FAO, which supports tissue repair and resolution of inflammation. Central regulators of these pathways include HIF-1α 31, which promotes glycolytic gene expression under hypoxic or inflammatory conditions, and AMPK, which favors catabolic metabolism and energy conservation during stress. The mTOR pathway, responsive to nutrient availability, supports anabolic metabolism and contributes to pro-inflammatory activation 32-34. This metabolic flexibility allows lung macrophages to adjust their function according to the tissue environment. While such adaptability is essential for maintaining homeostasis, it also provides a basis for dysregulation in chronic disease, where persistent environmental stressors can lock macrophages into maladaptive metabolic and inflammatory states.

## 3. Macrophage immunometabolism in chronic lung diseases

Chronic lung diseases such as asthma, COPD, and IPF are associated with persistent immune activation and progressive tissue remodeling. In each of these conditions, macrophages exhibit distinct metabolic profiles that reflect their adaptation to the local microenvironment. These disease-specific metabolic programs influence macrophage function and, in many cases, contribute directly to the progression of inflammation, tissue damage, or fibrosis.

### 3.1 Asthma

Asthma is a chronic airway disease characterized by type 2 inflammation, airway hyperresponsiveness, mucus overproduction, and structural remodeling 35, 36. In allergic asthma, AMs are exposed to a cytokine environment dominated by IL-4 and IL-13, which promote alternative (M2-like) activation. As a result, airway macrophages display increased expression of CD206 and other markers associated with IL-4/IL-13-mediated polarization, indicating a shift toward a tissue-repair and immunomodulatory phenotype 37. Notably, in mild allergic asthma, M2-polarized AMs can help resolve inflammation by engulfing apoptotic eosinophils and producing anti-inflammatory cytokines such as IL-10 38. However, in more severe forms of asthma, this resolution program is impaired. AMs lose their ability to clear apoptotic cells effectively and show reduced IL-10 production, leading to persistent inflammation and airway remodeling, including subepithelial fibrosis and smooth muscle hyperplasia 39. In severe asthma, pro-inflammatory macrophage subsets also emerge, such as those expressing interferon regulatory factor 5 (IRF5), suggesting considerable phenotypic heterogeneity within the macrophage pool 40. This diversity reflects a functional plasticity shaped by the chronic inflammatory microenvironment.

**Figure 3:**
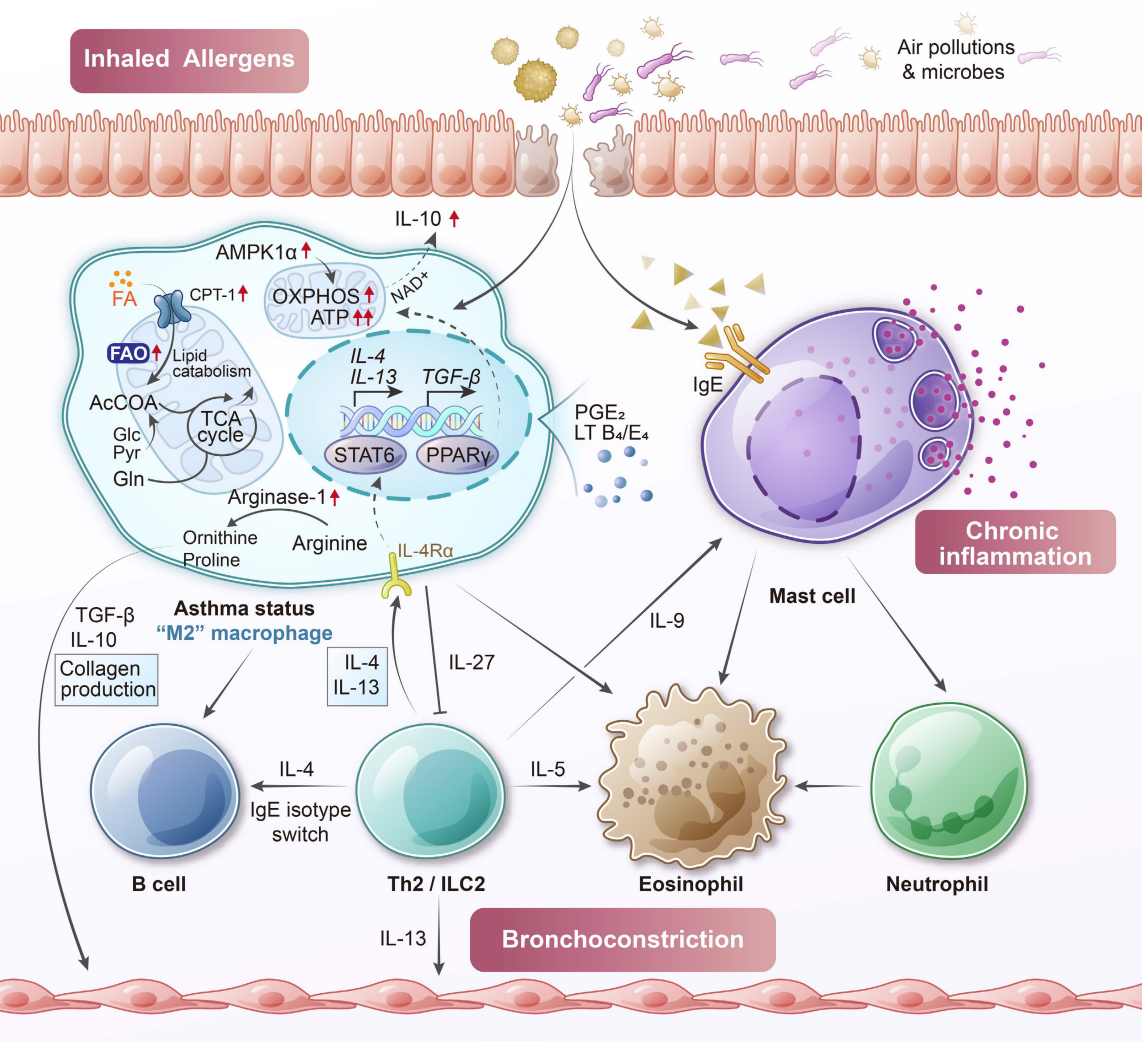
** Allergic asthma-associated macrophage metabolism.** In asthma, alveolar macrophages are exposed to a cytokine environment dominated by IL-4 and IL-13, which promote alternative (M2-like) activation and shift metabolic programming toward enhanced fatty acid oxidation. These macrophages express high levels of carnitine palmitoyl transferase 1 (CPT1) and arginase-1, supporting tissue repair functions but also contributing to subepithelial fibrosis and airway remodeling. Concurrently, elevated reactive oxygen species (ROS) production and disordered glycolytic activity reflect a broader metabolic imbalance. Asthmatic macrophages also display altered lipid mediator metabolism, producing excess leukotrienes while failing to generate sufficient pro-resolving eicosanoids such as 15-hydroxy eicosatetraenoic acid (15-HETE) and prostaglandin E_2_ (PGE_2_). These changes impair inflammation resolution, disrupt efferocytosis, and drive persistent airway inflammation. AcCoA, acetyl-coenzyme A; AMPK, adenosine monophosphate-activated protein kinase; NAD^+^, nicotinamide adenine dinucleotide (oxidized form).

The metabolic programming of macrophages in asthma reflects this skewed activation state (**Figure 3**). Chronic exposure to allergens and type 2 cytokines enhances mitochondrial oxidative metabolism in AMs. These cells exhibit increased FAO, as evidenced by elevated expression of carnitine palmitoyl transferase 1 (CPT1) and other enzymes involved in mitochondrial fatty acid uptake and β-oxidation 41. Such metabolic orientation supports the energy demands of M2-like functions, including matrix remodeling and secretion of fibrogenic mediators. Moreover, this FAO enhancement may represent a compensatory adaptation to the lipid-rich airway environment during inflammation 42, 43.

At the same time, asthmatic macrophages demonstrate increased oxidative stress. Elevated production of reactive oxygen species (ROS), such as hydrogen peroxide and superoxide, has been detected in AMs from asthmatic patients 44. This is accompanied by upregulation of oxidative stress response genes, including heme oxygenase-1 (HO-1), indicating sustained redox imbalance 45. ROS overproduction can contribute to epithelial injury 46, promote pro-inflammatory signaling 47, and enhance airway hyperresponsiveness. Importantly, this oxidative burden coexists with metabolic dysfunction in glycolysis pathways, suggesting a broader metabolic imbalance rather than a simple shift toward FAO 48.

Asthma is also associated with disruptions in macrophage lipid mediator metabolism 49. IL-4 and IL-13 stimulation enhances the production of pro-inflammatory eicosanoids, such as leukotriene B_4_ (LTB_4_), LTE_4_, and 15-hydroxyeicosatetraenoic acid (15-HETE) 50. These mediators promote bronchoconstriction, leukocyte recruitment, and mucus secretion. In contrast, the generation of anti-inflammatory lipid mediators, including prostaglandin E_2_ (PGE_2_) and 15-HETE, is diminished in asthmatic macrophages 51. This imbalance between pro-inflammatory and pro-resolving lipid mediators impairs efferocytosis and contributes to the perpetuation of airway inflammation 52. Furthermore, the expression of arginase-1 (Arg1) is increased in asthmatic macrophages 53, 54. This enzyme metabolizes L-arginine into ornithine and urea 55, 56, diverting substrate away from nitric oxide (NO) synthesis and instead facilitating polyamine and proline production 56, which support collagen synthesis and airway remodeling 57. The upregulation of Arg1, together with increased FAO, reflects a metabolic environment that favors tissue repair and matrix deposition but may also contribute to fibrosis if left unchecked 58-60.

Although some of these M2-associated features serve to limit inflammation and promote resolution, their persistence or dysregulation in chronic disease may result in pathological tissue remodeling 61, 62. In severe asthma, the regulatory capacity of AMs becomes compromised 39. These cells lose their ability to effectively clear apoptotic cells and dampen excessive inflammation, leading to a mixed phenotype that combines elements of M2-like metabolism with elevated oxidative stress and persistent cytokine production 63-65. Consistent with these alterations, biomarkers of macrophages activation are modified in asthmatic airway. AMs from asthmatic patients exhibit high pression of Arg1, reflecting an M2-skewed phenotype, and reduced production of PGE_2_. Additionally, levels of the chitinase-like protein YKL-40 (CHI3L1), secreted by macrophages, are elevated in both serum and bronchoalveolar lavage fluid of patients with severe asthma 66, 67.

The immunometabolic reprogramming of AMs in asthma involves enhanced FAO, increased ROS generation, and imbalanced eicosanoid synthesis. These changes promote both structural remodeling and chronic inflammation. The asthmatic environment pushes AMs into a metabolically active yet functionally imbalanced state. It is as if one foot is pressing the gas pedal, driven by increased ROS production and disordered glycolysis, while the other foot is on the brake, sustaining M2-like tissue-repair activity. This conflicting metabolic behavior prevents proper resolution of inflammation and contributes to ongoing tissue damage 63.

### 3.2 COPD

COPD is a chronic lung condition that is primarily caused by prolonged exposure to cigarette smoke 68. It is characterized by persistent airway inflammation, progressive airflow limitation, and destruction of alveolar structures, leading to emphysema 69. A hallmark of COPD is the accumulation of activated macrophages in the small airways and alveolar spaces. In smokers and individuals with COPD, the number of AMs in bronchoalveolar lavage fluid can increase by several folds 70. These macrophages contribute to disease progression by releasing pro-inflammatory cytokines, chemokines, and proteases 71. Among these, macrophage metalloelastases are directly implicated in elastin degradation and tissue destruction. Simultaneously, COPD AMs are functionally compromised in their host defense capacity. Multiple studies have demonstrated that COPD macrophages exhibit reduced phagocytic activity and impaired bacterial killing, which contributes to frequent microbial colonization and recurrent disease exacerbations 72.

A defining feature of the COPD lung microenvironment is the presence of high oxidative stress, which far exceeds that observed in asthma 73. Cigarette smoke introduces large quantities of exogenous free radicals into the lungs and stimulates resident macrophages to produce additional ROS 74. Compared to non-smokers, AMs from COPD patients release significantly higher levels of mitochondrial ROS, superoxide anion (O_2_^⁻^), and hydrogen peroxide (H_2_O_2_), indicating chronic oxidant exposure 75. This oxidative imbalance is further aggravated by insufficient antioxidant defenses in COPD macrophages. For example, the expression of glutamate-cysteine ligase, the rate-limiting enzyme in glutathione biosynthesis, is downregulated in these cells 76. As a result, the oxidant burden in the COPD lung remains high, not only damaging surrounding tissue structures but also impairing the function and viability of the macrophages themselves.

Mitochondrial dysfunction is a prominent feature of COPD AMs and is strongly linked to the sustained oxidative environment caused by cigarette smoke exposure 77. Persistent ROS accumulation leads to mitochondrial membrane depolarization and disruption of the electron transport chain 78. In COPD patient-derived AMs, studies have revealed a decreased mitochondrial membrane potential, along with significantly reduced maximal respiratory capacity and diminished spare respiratory reserve 74. Bioenergetic profiling further shows that while baseline glycolysis rates remain similar between healthy individuals and those with COPD, COPD macrophages exhibit substantially lower OXPHOS efficiency and increased proton leak 79. This indicates a population of mitochondria that are energetically inefficient and structurally compromised. Consequently, these cells cannot generate adenosine triphosphate (ATP) effectively through OXPHOS. Moreover, mitochondrial damage leads to greater leakage of ROS, forming a vicious cycle of oxidative stress. This bioenergetic dysfunction likely contributes to impaired phagocytosis. When mitochondria fail to provide sufficient ATP, or when excess mitochondrial ROS interferes with cellular signaling, macrophages become unable to efficiently engulf and eliminate bacteria. One study has demonstrated that the increase in mitochondrial ROS observed in COPD AMs is causally linked to their defective bacterial clearance 74.

In response to mitochondrial impairment, COPD macrophages may attempt to adapt their metabolism. Evidence supports a shift toward glycolytic metabolism, largely driven by HIF-1α 80, 81. Transcriptomic analyses of COPD AMs show increased expression of HIF-1α and its downstream targets, including the adenosine A_2_B receptor, a gene often upregulated under hypoxic or glycolytic conditions 82. These findings suggest that despite the presence of normal oxygen levels, COPD macrophages experience a “pseudo-hypoxic” state due to mitochondrial dysfunction and oxidative stress, which stabilizes HIF-1α and promotes a metabolic shift toward glycolysis. However, this glycolytic compensation may be insufficient to restore energy balance. The resulting accumulation of lactate and altered metabolite profiles could further contribute to inflammation and immune dysregulation.

In addition to these changes, COPD macrophages show abnormalities in nitrogen and arginine metabolism. Unlike AMs in healthy lungs, which produce minimal NO, COPD macrophages exhibit concurrent upregulation of inducible nitric oxide synthase (iNOS) and Arg1 83, 84. Elevated iNOS expression is particularly evident in patients with advanced disease or during exacerbations 85, 86. Although this might be expected to increase NO production, levels of exhaled NO in stable COPD are not elevated. One likely explanation is the excessive level of superoxide in the COPD lung, which rapidly reacts with NO to form peroxynitrite (ONOO^⁻^), a highly reactive nitrogen species (RNS) 83. Supporting this, increased nitrotyrosine staining, an indicator of peroxynitrite-mediated protein damage, has been detected in sputum macrophages from COPD patients and correlates with worsened lung function 87. Meanwhile, Arg1 is also induced by cigarette smoke and depletes intracellular L-arginine, the common substrate for both iNOS and Arg1 88. When L-arginine levels become limiting, iNOS becomes “uncoupled,” shifting its activity from NO production to superoxide generation 84. This favors additional peroxynitrite formation and intensifies nitrosative stress. Therefore, COPD macrophages experience combined oxidative and nitrosative stress, producing ONOO^⁻^ that damages proteins, lipids, and DNA, and contributing to sustained inflammation and cell death. The simultaneous expression of iNOS and Arg1 in these cells reflects a mixed M1/M2 polarization pattern, driven by the complex and dysregulated microenvironment in COPD lungs 83.

Iron metabolism adds another layer to the immunometabolic dysfunction of COPD macrophages 89. Cigarette smoke contains iron-rich particles and promotes alveolar microhemorrhages that release heme, leading to iron loading in AMs 90, 91. These macrophages often appear as hemosiderin-laden “siderophages” under histological analysis, indicating increased intracellular iron content 92. Excess iron promotes the Fenton reaction, in which hydrogen peroxide is converted into highly reactive hydroxyl radicals, thereby exacerbating oxidative injury 93, 94. Moreover, iron overload and the associated oxidative stress can trigger ferroptosis, a form of iron-dependent cell death, in lung cells. This is increasingly recognized in COPD and pulmonary fibrosis, as iron-catalyzed lipid peroxidation feeds into cell death and inflammation 95. Iron-loaded macrophages further propagate inflammation and tissue damage 96. In one study, reducing iron availability—either by iron chelation or dietary iron restriction—provided protection against cigarette smoke-induced lung injury in mice 97. These findings support a direct pathological role for iron overload in driving oxidative stress and macrophage dysfunction in COPD.

**Figure 4:**
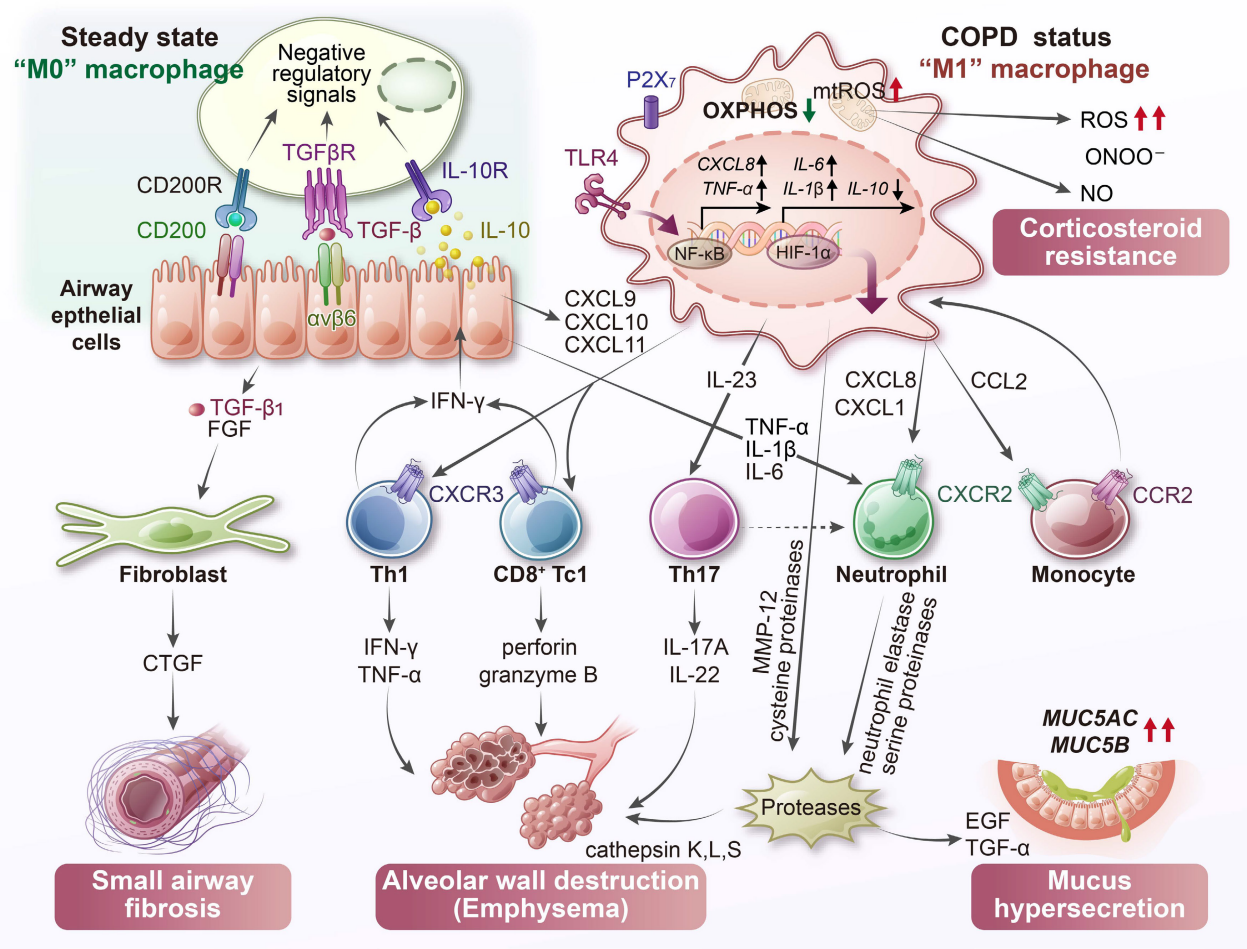
** Metabolic dysfunction in COPD macrophages.** In COPD, alveolar macrophages display mitochondrial impairment with reduced membrane potential and oxidative phosphorylation (OXPHOS) efficiency. Chronic exposure to cigarette smoke promotes iron accumulation and excessive ROS, fueling Fenton reactions and the formation of peroxynitrite (ONOO^⁻^). Concurrent stabilization of hypoxia-inducible factor-1α (HIF-1α), even under normoxic conditions, drives a metabolic shift toward glycolysis and enhances pro-inflammatory activity. These macrophages secrete a range of inflammatory mediators that orchestrate immune cell recruitment: CXCL9, CXCL10, and CXCL11 attract Th1 and Tc1 lymphocytes; CXCL8, CXCL1, and leukotriene B_4_ (LTB_4_) recruit neutrophils; and CCL2 mediates monocyte infiltration. The release of proteolytic enzymes, including matrix metalloproteinases (MMPs) and cathepsins, contributes to elastin degradation, synergizing with cytotoxic T cells to promote emphysema. Transforming growth factor-β_1_ (TGF-β_1_) released by macrophages also drives small airway fibrosis. Furthermore, the interaction between macrophage-derived ROS and nitric oxide (NO) leads to excessive ONOO^⁻^ generation, a process potentially linked to corticosteroid resistance in COPD. CTGF, connective tissue growth factor; FGF, fibroblast growth factor.

COPD induces profound metabolic disturbances in lung macrophages (**Figure 4**). Chronic smoke exposure and persistent ROS production lead to mitochondrial damage, disrupted energy metabolism, and imbalances in both reactive oxygen and nitrogen species. These stressors impair essential immune functions such as bacterial clearance and promote chronic inflammation and tissue destruction. Multiple inflammatory biomarkers reflect the macrophage-driven changes in COPD (**Table 2**). Sputum levels of IL-8 and matrix metalloproteinase (MMP)-9, partly derived from macrophages, are significantly elevated and correlate with neutrophilic inflammation as well as progressive decline in lung function 98. Likewise, sputum macrophages in COPD exhibit increased levels of 3-nitrotyrosine, a marker of peroxynitrite-mediated oxidative damage, and these elevations show an inverse correlation with FEV_1_% 87. Therapeutic strategies aimed at correcting macrophage metabolic dysfunction, such as restoring mitochondrial function or enhancing antioxidant capacity, hold promise for improving disease outcomes. Indeed, activation of the NRF2 (nuclear erythroid-related factor 2) antioxidant pathway in COPD macrophages has been shown to improve bacterial clearance 99, suggesting that immunometabolic correction can provide clinical benefit.

### 3.3 IPF

IPF is a chronic and progressive interstitial lung disease characterized by excessive fibrotic remodeling 100. This condition involves the accumulation of collagen and extracellular matrix within the lung parenchyma, which leads to architectural distortion and ultimately respiratory failure. The pathogenesis of IPF is believed to stem from aberrant wound-healing responses to unidentified lung injuries. These responses include repetitive alveolar epithelial cell damage and dysregulated activation of fibroblasts and myofibroblasts 101. Importantly, unlike asthma and COPD, IPF is not primarily an inflammatory disorder; broad anti-inflammatory or immunosuppressive therapies have shown little benefit and may even be harmful 102. Instead, the differentiation of fibroblasts into myofibroblasts is considered the central pathogenic event driving disease progression, as these cells directly mediate fibrotic remodeling of the lung. AMs play a critical supporting role by secreting pro-fibrotic mediators such as TGF-β and platelet-derived growth factor (PDGF), while also sustaining fibroblast activation; however, they are not the main effectors of scarring 103. In IPF lungs, AMs accumulate in fibrotic foci and exhibit an “M2-like” activation profile associated with wound healing. However, under persistent stimulation, this phenotype becomes pathogenic. These macrophages release large amounts of TGF-β and ROS, both of which directly stimulate myofibroblasts to produce collagen and thereby perpetuate fibrogenesis.

Recent studies suggest that AMs in IPF undergo global metabolic activation and become hypermetabolic cells. In contrast to the metabolically restrained phenotype of homeostatic AMs, IPF macrophages upregulate both glycolytic and oxidative metabolic pathways. In animal models of pulmonary fibrosis, such as bleomycin-induced lung injury, AMs demonstrate increased glucose uptake and a shift toward aerobic glycolysis, as evidenced by enhanced expression of key glycolytic enzymes and glucose transporters 104, 105. Consistent findings have been observed in humans: AMs from IPF patients display elevated expression of glucose transporter 1 (GLUT1) 105, indicating an increased capacity for glucose uptake 104. This is somewhat analogous to cancer cells in a tumor microenvironment, in which glycolysis supports the rapid generation of ATP and biosynthetic intermediates required for high secretory activity 106, 107. Moreover, glycolytic reprogramming can stabilize HIF-1α and enhance the production of pro-fibrotic cytokines like IL-1β in macrophages 13, further contributing to the fibrotic process.

**Figure 5:**
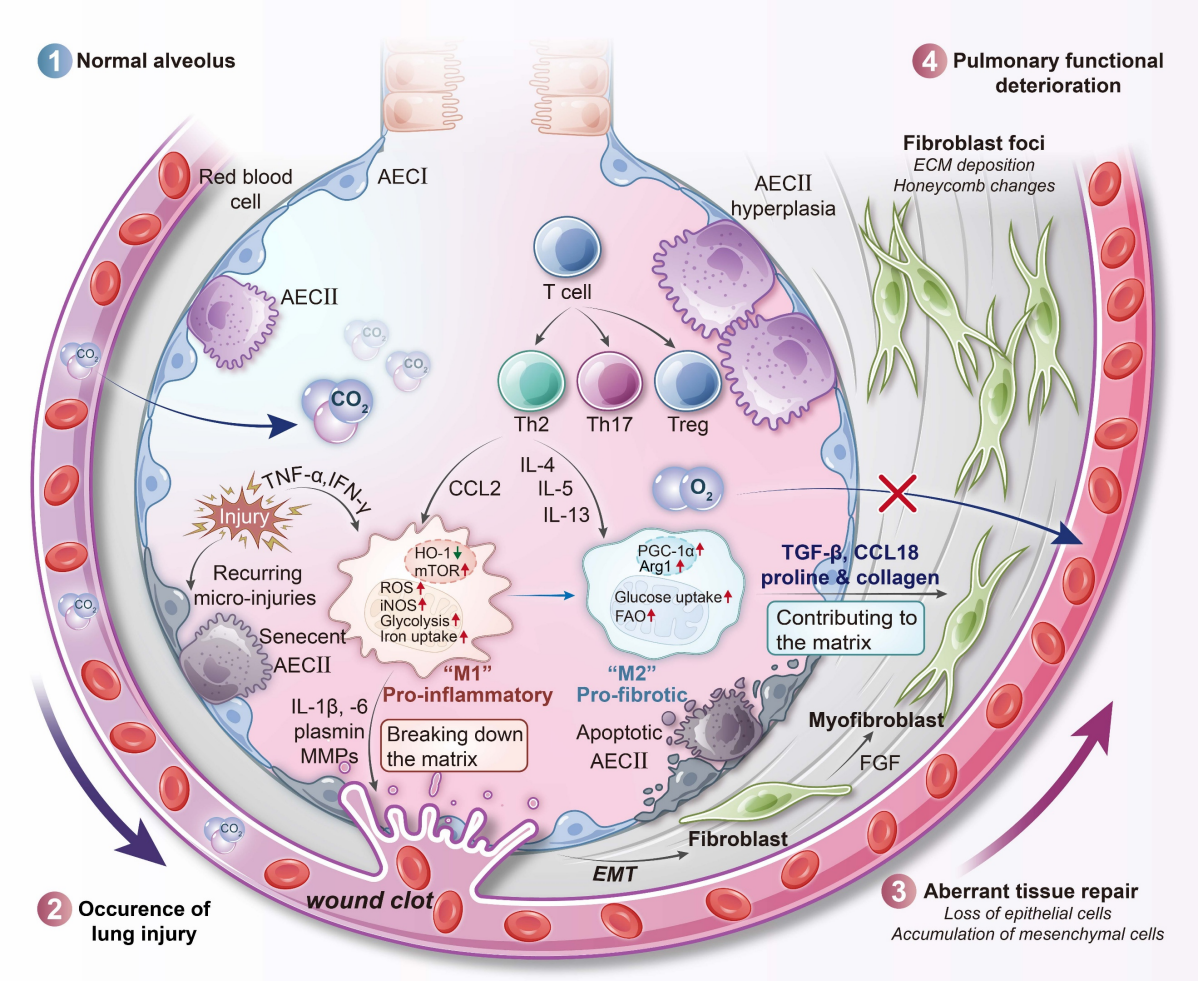
** Immunometabolic profile of IPF macrophages.** In IPF, alveolar macrophages exhibit distinct metabolic polarization that reflects the progression from early inflammation to late-stage fibrosis. Early in disease, macrophages may adopt a glycolysis-driven M1-like phenotype associated with pro-inflammatory cytokine production, including IL-1β. As fibrosis advances, these cells shift toward an M2-like metabolic profile characterized by enhanced fatty acid oxidation, upregulated PGC-1α signaling, and sustained TGF-β secretion. Concurrent mitochondrial calcium influx amplifies oxidative phosphorylation and ROS output. Suppression of the aconitate decarboxylase 1 (ACOD1)-itaconate pathway removes an anti-inflammatory regulatory node, enabling persistent macrophage activation. Together, these metabolic changes promote fibroblast activation, collagen deposition, and irreversible tissue remodeling. AEC, alveolar epithelial cell; ECM, extracellular matrix; EMT, epithelial-mesenchymal transition; HO-1, heme oxygenase 1.

In parallel, IPF AMs exhibit heightened FAO and increased mitochondrial respiration. Notably, studies in IPF patients have shown upregulated activity of the mitochondrial calcium uniporter and its downstream effector peroxisome proliferator-activated receptor γ coactivator 1-α (PGC-1α), a central regulator of mitochondrial biogenesis and FAO 108. Upregulation of CPT1, a key enzyme in FAO, has been observed in both IPF patient samples and bleomycin-induced fibrotic lungs, indicating a significant increase in FAO capacity 104, 108. Thus, IPF macrophages display a hybrid metabolic state, engaging in both aerobic glycolysis and oxidative metabolism simultaneously (**Figure 5**) 109. This state of enhanced metabolic flux likely reflects the energy demands of continuous secretion of growth factors, proteases, and ROS 110. By utilizing both glucose and fatty acids as energy substrates, these cells maintain a robust supply of ATP and biosynthetic precursors necessary for their profibrotic activity 111. However, this hypermetabolic state also leads to elevated mitochondrial ROS production, a known consequence of increased OXPHOS 112.

One metabolic checkpoint that appears disrupted in IPF macrophages is the itaconate pathway. Itaconate, produced by the mitochondrial enzyme immune-responsive gene 1 (IRG1, also known as ACOD1 [aconitate decarboxylase 1]), serves as a negative feedback regulator during macrophage activation 113. It inhibits succinate dehydrogenase activity in the TCA cycle, activates the NRF2 pathway, and reduces IL-1β production, thereby exerting anti-inflammatory effects. In IPF, this protective mechanism is compromised. Alveolar lining fluid from IPF patients contains significantly reduced levels of itaconate, and macrophages from fibrotic lungs exhibit decreased expression of ACOD1 compared to healthy controls 114.

Functionally, this loss of itaconate signaling shifts macrophages toward a more pro-inflammatory and pro-fibrotic phenotype. In mouse models, genetic deletion of *Acod1* exacerbates lung fibrosis after injury, while treatment with exogenous itaconate or derivatives such as 4-octyl itaconate reduces fibroblast activation and collagen deposition 114. These findings suggest that the absence of endogenous itaconate removes an important regulatory brake on TGF-β signaling and extracellular matrix production in IPF. The loss of this single metabolite contributes significantly to macrophage-driven fibrogenesis.

Consistent with their hyperactivated metabolic profile, IPF macrophages also generate large amounts of ROS and RNS 115. Elevated levels of *Rac1*, a small GTPase that activates NADPH oxidase, have been observed in IPF macrophages, driving superoxide production 116, 117. In IPF lungs, the combined effects of NADPH oxidase-derived ROS and mitochondrial ROS from upregulated OXPHOS create a highly oxidizing milieu. Simultaneously, these macrophages express iNOS, and inflammatory stimuli in IPF are capable of inducing robust iNOS activity. As a result, NO and superoxide co-exist within the same microenvironment and react to form peroxynitrite (ONOO^⁻^). Elevated levels of ONOO^⁻^ and its oxidative byproducts, such as nitrotyrosine, have been detected in IPF patient-derived AMs 118. In the bleomycin model of fibrosis, AMs show increased levels of superoxide, NO, and ONOO^⁻^ during the active fibrotic phase 119. These reactive species can activate latent TGF-β and induce alveolar epithelial cell apoptosis, thereby driving further fibrosis.

Iron dysregulation in macrophages represents another immunometabolic abnormality in IPF. Similar to findings in COPD, IPF lungs frequently contain hemosiderin-laden AMs, suggesting chronic microhemorrhages or enhanced iron uptake. These iron-loaded macrophages contribute to fibrogenesis through several mechanisms 120. Interestingly, the proportion of AMs expressing transferrin receptor (CD71) is reduced in IPF lungs, which may indicate a compensatory mechanism in response to intracellular iron overload 121. The result is increased levels of extracellular, transferrin-bound iron in the fibrotic niche. Free iron can catalyze redox reactions that generate hydroxyl radicals via the Fenton reaction, promoting additional oxidative damage. Moreover, iron accumulation promotes macrophage polarization toward a pro-fibrotic phenotype 122. Iron-rich AMs secrete ROS and pro-fibrotic cytokines and can directly stimulate fibroblast proliferation and myofibroblast differentiation 123. Experimental reduction of iron availability through dietary restriction or iron chelation has been shown to ameliorate lung fibrosis in animal models 124, 125, underscoring the pathological relevance of iron-driven macrophage activation in IPF. Emerging evidence also links iron overload in IPF to ferroptosis 95, 126. In late-stage fibrotic lungs, substantial iron deposition and oxidative lipid damage have been observed, which may promote death of alveolar epithelial cells and even macrophages via ferroptosis 124. This ferroptotic injury is thought to further amplify profibrotic signaling by releasing damage-associated molecular patterns (DAMPs) and activating neighboring fibroblasts 95. Thus, the dysregulated iron homeostasis in IPF not only accelerates ROS/RNS generation but also intersects with novel pathogenic mechanisms like ferroptosis, creating self-perpetuating cycles of injury and fibrosis.

AMs in IPF are metabolically reprogrammed into highly active, fibrosis-promoting cells. They simultaneously engage glycolysis and FAO, maintain high levels of ATP and ROS production, and lose regulatory mechanisms such as itaconate signaling. Iron accumulation and sustained oxidative/nitrosative stress further amplify their pathogenic activity. This metabolic polarization of macrophages plays a central role in IPF progression and represents a promising target for therapeutic intervention.

### 3.4 Comparative perspective

Despite the distinct metabolic fingerprints of asthma, COPD, and IPF, there are unifying themes in how macrophage metabolism is dysregulated. Each condition drives macrophages toward a particular metabolic bias that reinforces the predominant pathology. In asthma, a cytokine-rich, type 2 environment pushes macrophages toward oxidative metabolism and lipid utilization, resembling an M2-like profile that supports tissue remodeling but, when unchecked, contributes to fibrosis and impaired resolution. In COPD, chronic oxidative stress and hypoxia-inducible signals enforce a glycolytic, Warburg-like metabolism (akin to an M1 state) but in the context of mitochondrial dysfunction, resulting in energy failure, persistent inflammation, and tissue injury. IPF macrophages uniquely combine these metabolic programs, exhibiting concurrent glycolysis and OXPHOS to fuel relentless fibrogenesis (**Table 3**).

The interplay between glycolysis and fatty acid oxidation is context-dependent. Inflammatory macrophages typically downregulate FAO as they upregulate glycolysis, as seen in acute M1 activation, whereas pro-resolving macrophages favor FAO and dampen glycolysis, consistent with classic M2 polarization. However, in chronic disease microenvironments, these pathways can co-exist or cycle dynamically, indicating that macrophages can flexibly rewire their metabolism when faced with complex stimuli. Key regulators such as HIF-1α and PPAR-γ act in opposition to fine-tune this balance: HIF-1α activation, even under normoxic conditions, promotes glycolysis and can inhibit mitochondrial respiration, whereas PPAR-γ, upregulated by signals like IL-4 and GM-CSF, enhances lipid uptake and oxidation while antagonizing inflammatory glycolysis. In diseases like IPF, both arms are engaged, suggesting that continuous injury signals override the conventional glycolysis-versus-FAO toggle.

Understanding these metabolic interactions is essential, as targeting a single pathway such as glycolysis may cause compensatory shifts in another (FAO), so successful therapies might need to address the metabolic network as a whole. In the next section, we discuss how these insights are being leveraged to design interventions that recalibrate macrophage metabolism across different lung diseases.

## 4. Therapeutic targeting of macrophage immunometabolism

Given the central role of macrophage metabolic reprogramming in chronic lung diseases, a number of therapeutic strategies are being explored with the aim of reconfiguring macrophage metabolism toward a more balanced and health-promoting state. These interventions seek to restore the physiological equilibrium between pro-inflammatory and reparative macrophage functions, or to enhance antimicrobial capacity through metabolic modulation. Several approaches have demonstrated encouraging results in preclinical or early-phase clinical studies.

### 4.1 Antioxidants and NRF2 activators

One potential therapeutic approach centers on reducing oxidative stress within macrophages, with the goal of alleviating tissue injury and restoring cellular function. *N*-acetylcysteine, a precursor of glutathione, has been tested as an oral antioxidant therapy in IPF, as exemplified by the PANTHER trial 127. Although this trial did not demonstrate significant improvements in lung function, and the concomitant use of prednisone and azathioprine in that treatment arm actually worsened outcomes, *N*-acetylcysteine and other thiol-based antioxidants remain of interest as strategies to replenish intracellular glutathione in diseases such as COPD or cystic fibrosis, where they may mitigate oxidant-mediated macrophage dysfunction. More targeted strategies aim to activate the NRF2 pathway, a key regulator of antioxidant defense gene expression. Sulforaphane, a naturally occurring NRF2 agonist found in cruciferous vegetables, has been shown to enhance phagocytosis in AMs isolated from COPD patients by upregulating antioxidant responses 128. These findings suggest that NRF2 activation can reverse certain functional deficits in COPD macrophages. Similarly, dimethyl fumarate, a pharmacologic NRF2 activator currently used for multiple sclerosis 129, has been reported to shift macrophages toward an anti-inflammatory phenotype 130. Collectively, agents that enhance the expression of HO-1, glutathione biosynthesis enzymes, and other antioxidant mediators constitute a mechanistically grounded approach to suppressing the ROS/RNS-driven inflammatory cycle characteristic of chronic lung diseases.

### 4.2 Metabolic pathway inhibitors (glycolysis and FAO)

Because disease-associated macrophages in asthma and IPF exhibit concurrent upregulation of glycolysis and FAO, selective inhibition of these pathways may attenuate pathogenic macrophage activation 41, 131, 132. 2-deoxy-D-glucose, a competitive inhibitor of glycolysis, has been shown in murine models of allergic asthma to prevent the pathological glycolytic shift in AMs 133. Treatment with 2-deoxy-D-glucose reduced airway inflammation and hyperresponsiveness, suggesting that limiting aerobic glycolysis can downregulate cytokine production in asthma-associated AMs 134. Conversely, etomoxir, an inhibitor of CPT1 that blocks mitochondrial FAO, has also shown beneficial effects in asthma models. Administration of etomoxir in mice reduced airway hyperreactivity, potentially by impairing the FAO-dependent alternative activation of AMs (“M2”) 41. Similar logic may apply to IPF, where inhibition of glycolysis or FAO may disrupt the hypermetabolic and pro-fibrotic programming of macrophages 135. However, systemic inhibition of core metabolic pathways poses significant risks, given their essential roles in all cell types. Therefore, careful control of drug dosage and delivery route is critical. Inhaled administration of low-dose 2-deoxy-D-glucose or etomoxir may enable local metabolic reprogramming of pulmonary macrophages with reduced systemic exposure and toxicity. Early studies support this idea, but clinical trials will be needed to confirm safety and efficacy in patients.

### 4.3 AMPK and mTOR modulators

The AMPK and mTOR pathways serve as central metabolic regulators that determine the balance between catabolic and anabolic processes. Activation of AMPK favors oxidative metabolism and promotes cellular resilience to stress, while mTOR activation supports glycolysis, protein synthesis, and pro-inflammatory cytokine production. Metformin, a well-characterized AMPK activator, has attracted attention for its potential to reprogram macrophage metabolism toward an anti-inflammatory state. *In vitro*, metformin enhances FAO and improves mitochondrial function in macrophages, which may support anti-inflammatory and autophagic phenotypes 136. In models of lung fibrosis, metformin has been shown to activate AMPK and concurrently inhibit TGF-β signaling in both macrophages and myofibroblasts, thereby reducing collagen deposition 137, 138. Nonetheless, translation to clinical benefit remains uncertain. A post hoc analysis of IPF patients receiving metformin did not demonstrate a clear therapeutic advantage, although patient heterogeneity and dosing variability could have influenced outcomes 139. On the other hand, pharmacological inhibition of mTOR, such as with rapamycin, may suppress macrophage activation by reducing glycolytic flux and inflammatory cytokine production 140. mTORC1 promotes protein synthesis and IL-1β translation in classically activated (“M1”) macrophages. Preclinical studies in COPD models have suggested that mTOR inhibition may reduce airway inflammation, but safety and long-term efficacy remain to be established 141, 142. Altogether, precise tuning of macrophage metabolic sensors—enhancing AMPK activity while suppressing mTOR—offers a rational framework for therapeutic intervention but necessitates targeted approaches to avoid adverse systemic effects 3, 143.

### 4.4 Lipid mediator and iron homeostasis interventions

Another promising therapeutic strategy targets the metabolic outputs of activated macrophages, particularly the imbalance between pro-inflammatory and pro-resolving lipid mediators 144. In asthma, overproduction of LTs by macrophages contributes to bronchoconstriction and airway inflammation 145. Pharmacologic agents that block LT synthesis (e.g., 5-lipoxygenase inhibitors such as zileuton) or LT receptor signaling (e.g., montelukast, a CysLT_1_ antagonist) are already in clinical use for symptom control 145. Beyond blocking harmful lipid mediators, supplementing beneficial, pro-resolving eicosanoids offers an alternative therapeutic angle. For example, 15-HETE, an anti-inflammatory lipid molecule, is deficient in asthmatic macrophages. In animal studies, exogenous delivery of 15-HETE inhibited LT production and improved airway responsiveness 146. Likewise, specialized pro-resolving mediators such as lipoxin A_4_ and resolvin D1—both derived from Ω-3 fatty acids—have been shown to reduce neutrophilic inflammation and enhance clearance of cellular debris in models of asthma and cystic fibrosis 147, 148.

Iron homeostasis also plays a crucial role in macrophage immunometabolism, particularly in COPD and IPF, where macrophages often accumulate excess intracellular iron 89, 94. Chelating agents such as deferoxamine and deferasirox can reduce iron availability and may be delivered via inhalation to selectively target iron-laden AMs 149. In murine models, dietary iron restriction or iron chelation protected against cigarette smoke-induced emphysema, suggesting that limiting iron burden may reduce macrophage-driven oxidative damage 150. Additional strategies include increasing macrophage expression of iron-export proteins such as ferroportin, or enhancing iron uptake via transferrin receptor modulation. These approaches aim to restore iron handling and mitigate iron-catalyzed oxidative injury 151. By reducing iron-driven ROS production, such interventions could also lower the risk of ferroptosis in lung tissues, a mechanism implicated in both pulmonary fibrosis and emphysema 126.

### 4.5 Targeted drug delivery to lung macrophages

A recurring challenge in macrophage-targeted metabolic therapy is achieving cell-type specificity without systemic toxicity 152. To this end, a variety of targeted delivery systems are under development that aim to direct therapeutic agents specifically to AMs. These systems leverage the phagocytic capacity of macrophages to preferentially uptake engineered carriers, such as nanoparticles or microspheres 153. Inhalable formulations, including liposome-encapsulated drugs, have demonstrated preferential uptake by AMs 154. For instance, in the context of tuberculosis, aerosolized rifampicin-loaded microspheres have been successfully used to deliver antibiotics directly into AMs 155. Similar strategies may be adapted for metabolic modulation in chronic lung diseases. Nanoparticles carrying agents such as 2-deoxy-D-glucose, metformin, or antioxidants can be delivered via inhalation to achieve intracellular release within AMs. Surface modifications such as mannose coating further increase uptake efficiency by targeting the mannose receptor expressed on AMs 156.

Another promising modality involves inhaled nucleic acid therapeutics. Aerosolized small interfering RNAs (siRNAs) or antisense oligonucleotides can be designed to silence specific metabolic genes in AMs 157, such as HIF-1α or IRG1. This approach allows for selective attenuation of pathogenic signaling within pulmonary macrophages while minimizing effects on other cell types. In preclinical studies, combination therapies, such as co-delivery of iron chelators and nitrated fatty acids that activate anti-inflammatory pathways, have been shown to reprogram AMs toward a less injurious phenotype 153, 158.

Overall, these therapeutic strategies reflect a fundamental shift from broad immunosuppression (e.g., steroids) toward selective modulation of macrophage metabolic states. Their objective is no longer merely to suppress inflammation, but rather to restore physiological macrophage function by correcting underlying metabolic defects. This involves facilitating the resolution of inflammation, enhancing tissue repair, and improving pathogen elimination. While some strategies have demonstrated efficacy in animal models, their translation to human patients presents challenges. The redundancy of metabolic pathways means that macrophages might compensate for the inhibition of one pathway by upregulating others, and patient heterogeneity implies that a one-size approach may not fit all. Nevertheless, this customized regulation of macrophage immunometabolism holds great promise. It targets the core functional alterations of macrophages in chronic lung diseases, potentially restoring their capacity to promote homeostasis and eliminate pathogens, rather than simply shutting them down. Future inhaled therapies or smart drugs targeting macrophage metabolism could become adjuncts or alternatives to existing treatments for asthma, COPD, and IPF.

## 5. Conclusion and future directions

Over the past decade, significant advances have deepened our understanding of how metabolic adaptation shapes macrophage function in the lung. AMs exhibit remarkable metabolic plasticity: they can shift between OXPHOS, glycolysis, and lipid metabolism to meet context-dependent demands. In chronic lung diseases such as asthma, COPD, and IPF, this plasticity is pathologically reprogrammed, resulting in maladaptive phenotypes that perpetuate inflammation, tissue remodeling, or fibrosis. Each disease imposes a distinct metabolic signature on AMs. In asthma, macrophages are skewed toward fatty acid metabolism and lipid mediator imbalance. In COPD, iron overload, mitochondrial dysfunction, and HIF-1α-driven glycolysis impair microbial clearance while promoting oxidative stress. In IPF, macrophages display concurrent upregulation of glycolysis and FAO, coupled with loss of anti-inflammatory metabolites such as itaconate.

Despite these disease-specific profiles, a unifying theme is evident: chronic perturbations in immunometabolism underlie macrophage dysfunction and disease persistence. Targeting macrophage metabolism presents an opportunity to restore homeostatic function without broadly suppressing immune responses. Preclinical data suggest that modulating key metabolic pathways, such as glycolysis, FAO, antioxidant defense, and iron handling, can shift macrophages away from pro-inflammatory or pro-fibrotic states. Moreover, early efforts in drug delivery platforms, including inhalable nanoparticles and macrophage-specific carriers, have demonstrated potential for selective and localized intervention.

A major challenge lies in the heterogeneity of macrophage populations within the lung. Single-cell and spatial transcriptomic studies have begun to reveal distinct metabolic phenotypes among macrophage subsets in both health and disease 159. To move beyond correlation, these insights must be integrated with direct metabolic measurements and functional models that capture human disease contexts. Lung organoids, precision-cut lung slices, and humanized mouse models may serve as critical platforms to test causal links between metabolic rewiring and disease progression.

Future therapies will require not only effective metabolic targets but also precise patient selection. Identifying metabolic biomarkers that reflect macrophage dysfunction could support personalized treatment strategies and improve clinical trial design. Ultimately, by restoring balanced immunometabolism, it may be possible to re-establish the macrophage's protective functions and interrupt the self-sustaining cycles of inflammation and injury that characterize chronic lung disease.

In conclusion, macrophage immunometabolism offers both a conceptual and mechanistic framework for comprehending chronic lung diseases at the cellular level. It also paves the way for a promising therapeutic approach that targets underlying functional impairments rather than merely addressing downstream symptoms. By correcting metabolic imbalance, it may be possible to restore macrophage homeostatic roles in host defense, resolution, and repair. Continued research at the intersection of immunology, metabolism, and respiratory biology will be key to turning this potential into clinical reality.

## Figures and Tables

**Table 1:** Functional plasticity and metabolic regulation in macrophage subsets.

	M1 [LPS(+IFN-γ)]	M2 [IL-4]
Stimuli	Classical activation: LPS, IFN-γ, TNF-α, GM-CSF	Alternative activation: IL-4, IL-13, IL-10, TGF-β, IL-33, IL-21
Markers	Surface: CD80/86, MHC-Ⅱ Intracellular: iNOS, IRF5, STAT1, NF-κB, HIF-1α	Surface: CD163, CD209, CXCR1, CXCR2, Dectin-1 Intracellular: Arginase 1, IRF4, STAT6, PPARγ
Function	Pro-inflammatory defense, Phagocytic, Microbial killing, Tissue damage, Anti-cancer immunity, Host defense	Anti-inflammatory, Wound healing, Efferocytosis, Hypersensitive response, Angiogenesis, Matric and tissue remodeling, Tumor progression and metastasis
Secretion	Cytokines: IL-1β, IL-6, IL-12, IL-23, TNF-α Chemokines: CXCL9, CXCL10, CXCL11, MIP-1α (CCL3) Free radicals: ROS, iNOS, NO	Cytokines: IL-10, IL-1RII, IL-1RA Chemokines: CCL17, CCL18, CCL22, CCL24 Growth factors: TGF-β_1_, VEGF, PDGF, EGF
Glycolysis	Increased glycolytic flux Lactate accumulation HIF-1α-induced production of pro-inflammatory cytokines (IL-1β, etc.)	Dispensable when OXPHOS is intact Glycolysis produces pyruvate to fuel TCA cycle
TCA cycle	Broken in two places: after citrate and after succinate	An intact TCA cycle Replenished with FAO and glutamine metabolism
OXPHOS	Dysfunctional OXPHOS and ETC Increased ROS generation	Increased mitochondrial biogenesis and respiratory capacity PGC1β-induced gene expression
PPP	Induced and required for ROS generation via NADPH oxidase, NO production, and nucleotide and protein synthesis	Not required/suppressed by the sedoheptulose kinase CARKL
Fatty acid metabolism	Increased lipid synthesis SREBP-induced gene expression	Increased fatty acid β-oxidation (FAO) STAT6- and PPARγ-induced gene expression
Amino acid metabolism	Arginine is converted to NO and citrulline by iNOS Glutamine metabolism regulates trained innate immunity	Arginase-1 metabolizes arginine to generate ornithine and urea Glutamine is essential for M2 polarization
Iron metabolism	Ferritin (iron storage)	Ferroportin (iron export)

**Abbreviations:** M1, classically activated macrophages; M2, alternatively activated macrophages; LPS, lipopolysaccharide; TNF-α, Tumor necrosis factor-α; GM-CSF, granulocyte-macrophage colony-stimulating factor; TGF-β, transforming growth factor-β; MHC, major histocompatibility complex class; iNOS, inducible nitric oxide synthase; IRF, interferon regulatory factor; STAT, signal transducer and activator of transcription; NF-κB, nuclear factor-κB; PPAR, peroxisome proliferator activated receptor; ROS, reactive oxygen species; VEGF, vascular endothelial growth factor; PDGF, platelet derived growth factor; EGF, epidermal growth factor; FAO, fatty acid β-oxidation; PGC-1β, PPARγ coactivator 1β; TCA, tricarboxylic acid; OXPHOS, oxidative phosphorylation; ETC, electron transport chain; PPP, pentose phosphate pathway.

**Table 2:** Representative biomarkers of macrophage activation and metabolic dysfunction in chronic lung diseases.

Disease	Biomarker	Source	Macrophage-derived?	Clinical Relevance
Asthma	Arginase-1	BALF macrophage	Yes (M2 macrophage)	Promotes collagen synthesis, airway fibrosis
	YKL-40 (CHI3L1) 67	Serum/BALF	Yes (macrophages, others)	Correlates with asthma severity and airway remodeling
	FeNO 160	Breath	Partially (epithelium via iNOS; modulated by Arg1)	Elevated in allergic (type 2) asthma; indicates airway eosinophilic inflammation; influenced by macrophage arginase activity
	LTB_4_	Sputum/BALF	Yes (macrophages, neutrophils)	Increased in asthmatic airways; contributes to bronchoconstriction and inflammation
	15-HETE & Lipoxin A4	BALF	Yes (macrophages)	Reduced in asthma; anti-inflammatory and pro-resolving lipid mediators
	CCL17 (TARC)	BALF	Yes	Macrophage-secreted chemokine; eosinophil/T cell recruitment
	Periostin	Serum	No	Biomarker of type 2 inflammation
COPD	CXCL8 (IL-8)	Sputum	Yes (macrophages, epithelium)	Markedly elevated in COPD sputum; drives neutrophil recruitment
	MMP-9, MMP-12	BALF/Sputum	Yes (macrophages, neutrophils)	Promote elastin degradation and emphysema progression
	3-Nitrotyrosine	Sputum cells	Yes (macrophages)	Reflects peroxynitrite (ONOO^-^)-mediated oxidative stress
	Hemosiderin 161	BALF macrophages	Yes (indicative)	Indicates iron overload and oxidative injury
	SP-A, SP-D 162	Serum	No	General marker of lung injury and exacerbations
IPF	CCL18/PARC 163, 164	Serum, BALF	Yes (M2 macrophages)	Reflects profibrotic M2 activation; predicts poor survival and faster disease progression
	YKL-40 (CHI3L1)	Serum, BALF	Yes (macrophages, others)	Associated with fibrosis and matrix remodeling
	MMP-7 165	Serum	Partially (epithelium, macrophages)	Indicates active epithelial injury and remodeling
	KL-6 (MUC1) 164, 166, 167	Serum	No (alveolar type Ⅱ cells)	Widely used diagnostic and prognostic biomarker for ILDs
	SP-D 164, 168	Serum	No	Reflect epithelial injury and surfactant disturbance
	Reduced itaconate (ACOD1 product) 114	BALF macrophages	Yes	Loss of anti-inflammatory metabolite regulation; linked to persistent activation
	CD44 169	BALF/lung tissue	Partially (macrophages, fibroblasts)	Regulates macrophage-fibroblast crosstalk; associated with fibrosis progression
	CX3CL1 (fractalkine) 170	Serum, BALF	Yes (Endothelium, macrophages)	Potential diagnostic biomarker; correlates with IPF progression
	S100A8/A9/A12 (S100 family) 171	Serum, BALF	Partially (macrophages, neutrophils)	Associated with inflammation, oxidative stress, and fibrogenesis

**Abbreviations:** BALF, bronchoalveolar lavage fluid; FeNO, fractional exhaled nitric oxide; LTB_4_, leukotriene B4; 15-HETE, 15-hydroxyeicosatetraenoic acid; CCL17, chemokine (C-C motif) ligand 17; TARC, thymus and activation-regulated chemokine; MMP, matrix metalloproteinase; SP, surfactant protein; CCL18/PARC, pulmonary and activation-regulated chemokine; KL-6, Krebs von den Lungen-6, a mucin 1 glycoprotein; ILDs, interstitial lung diseases; ACOD1, aconitate decarboxylase 1.

**Table 3:** Comparative metabolic reprogramming of macrophages in chronic lung diseases.

Feature	Asthma	COPD	IPF
Dominant phenotype	M2-like, but dysfunctional in severe disease	Mixed M1/M2, functionally impaired	Hypermetabolic, profibrotic
Primary metabolic shift	↑ FAO, ↑ arginase activity, altered lipid mediator metabolism	Mitochondrial dysfunction → ↑ glycolysis (pseudo-hypoxia), ↓ OXPHOS	↑ Glycolysis + ↑ FAO (hybrid state)
ROS/RNS balance	↑ ROS, impaired antioxidant defenses	Excessive ROS + peroxynitrite (ONOO^-^) due to iNOS/Arg1 co-expression	↑ ROS + RNS, mitochondrial and NADPH oxidase-derived
Iron metabolism	Mild changes, not central	Iron overload, siderophages, Fenton reaction	Iron overload, CD71^low^ AMs, ferroptosis link
Regulatory metabolites	Imbalanced lipid mediators (↓PGE2, ↓ resolvins, ↑ leukotrienes)	↓ Glutathione synthesis, impaired antioxidant capacity	↓ Itaconate (ACOD1), loss of anti-inflammatory brake
Crosstalk with other cells	Eosinophils (impaired efferocytosis), epithelial cells	Neutrophils, T cells, epithelial damage	Fibroblasts/myofibroblasts (profibrotic loop)
Clinical implication	Contributes to airway remodeling, persistent inflammation	Drives impaired host defense, steroid resistance, emphysema	Sustains fibroblast activation, irreversible fibrosis

## Abbreviations

AM: alveolar macrophage
AMPK: AMP-activated protein kinase
Arg1: arginase-1
ATP: adenosine triphosphate
CARKL: carbohydrate kinase-like protein
COPD: chronic obstructive pulmonary disease
CPT1: carnitine palmitoyl transferase 1
FAO: fatty acid oxidation
GLUT: glucose transporter
GM-CSF: granulocyte-macrophage colony-stimulating factor
HETE: hydroxy eicosatetraenoic acid
HIF: hypoxia-inducible factor
HO-1: heme oxygenase-1
IL: interleukin
IM: interstitial macrophage
iNOS: inducible nitric oxide synthase
IPF: idiopathic pulmonary fibrosis
IRF: interferon regulatory factor
IRG: immune-responsive gene
LPS: lipopolysaccharide
LT: leukotriene
mTOR: mechanistic target of rapamycin
NO: nitric oxide
NRF2: nuclear erythroid-related factor 2
OXPHOS: oxidative phosphorylation
PDGF: platelet-derived growth factor
PG: prostaglandin
PPAR-γ: peroxisome proliferator-activated receptor-γ
RNS: reactive nitrogen species
ROS: reactive oxygen species
TCA: tricarboxylic acid
TGF: transforming growth factor
